# Spine Stereotactic Body Radiotherapy to Three or More Contiguous Vertebral Levels

**DOI:** 10.3389/fonc.2022.912804

**Published:** 2022-06-08

**Authors:** Khaled Dibs, Dukagjin M. Blakaj, Rahul N. Prasad, Alexander Olausson, Eric C. Bourekas, Daniel Boulter, Ahmet S. Ayan, Eric Cochran, William S. Marras, Prasath Mageswaran, Evan Thomas, Hyeri Lee, John Grecula, Raju R. Raval, Ehud Mendel, Thomas Scharschmidt, Russell Lonser, Arnab Chakravarti, James B. Elder, Joshua D. Palmer

**Affiliations:** ^1^ Department of Radiation Oncology, The James Cancer Hospital at the Ohio State University Wexner Medical Center, Columbus, OH, United States; ^2^ Department of Radiology, The James Cancer Hospital at the Ohio State University Wexner Medical Center, Columbus, OH, United States; ^3^ Spine Research Institute, Department of Biomedical Engineering, College of Engineering, The Ohio State University, Columbus, OH, United States; ^4^ Department of Neurosurgery, Yale University School of Medicine, New Haven, CT, United States; ^5^ Department of Orthopedic Surgery, The James Cancer Hospital at the Ohio State University Wexner Medical Center, Columbus, GA, United States; ^6^ Department of Neurosurgery, The James Cancer Hospital at the Ohio State University Wexner Medical Center, Columbus, GA, United States

**Keywords:** local control, multilevel, postoperative, radiosurgery, spine metastases, stereotactic body radiation therapy, stereotactic radiotherapy, toxicity

## Abstract

**Background:**

With survival improving in many metastatic malignancies, spine metastases have increasingly become a source of significant morbidity; achieving durable local control (LC) is critical. Stereotactic body radiotherapy (SBRT) may offer improved LC and/or symptom palliation. However, due to setup concerns, SBRT is infrequently offered to patients with ≥3 contiguous involved levels. Because data are limited, we sought to evaluate the feasibility, toxicity, and cancer control outcomes of spine SBRT delivered to ≥3 contiguous levels.

**Methods:**

We retrospectively identified all SBRT courses delivered between 2013 and 2019 at a tertiary care institution for postoperative or intact spine metastases. Radiotherapy was delivered to 14–35 Gy in 1–5 fractions. Patients were stratified by whether they received SBRT to 1–2 or ≥3 contiguous levels. The primary endpoint was 1-year LC and was compared between groups. Factors associated with increased likelihood of local failure (LF) were explored. Acute and chronic toxicity was assessed. In-depth dosimetric data were collected.

**Results:**

Overall, 165 patients with 194 SBRT courses were identified [54% were men, median age was 61 years, 93% had Karnofsky Performance Status (KPS) ≥70, and median follow-up was 15 months]. One hundred thirteen patients (68%) received treatment to 1–2 and 52 to 3–7 (32%) levels. The 1-year LC was 88% (89% for 1–2 levels vs. 84% for ≥3 levels, p = 0.747). On multivariate analysis, uncontrolled systemic disease was associated with inferior LC for patients with ≥3 treated levels. No other demographic, disease, treatment, or dosimetric variables achieved significance. Rates of new/progressive fracture were equivalent (8% vs. 9.5%, p = 0.839). There were no radiation-induced myelopathy or grade 3+ acute or late toxicities in either group. Coverage of ≥95% of the planning target volume with ≥95% prescription dose was similar between groups (96% 1–2 levels vs. 89% ≥3 levels, p = 0.078).

**Conclusions:**

For patients with ≥3 contiguous involved levels, spine SBRT is feasible and may offer excellent LC without significant toxicity. Prospective evaluation is warranted.

## Introduction

Spine metastases are a common source of morbidity for patients with cancer, as resultant pain and neurologic dysfunction can significantly diminish quality of life (1). Thus, radiation therapy (RT) ± surgery is frequently indicated for improved local control (LC) and/or symptom palliation (1). Historically, conventional external beam radiation therapy (EBRT) was considered the standard of care for patients requiring palliative RT. However, stereotactic body radiotherapy (SBRT) has recently been shown to offer significantly improved LC and motor function, particularly for patients with radioresistant tumor histologies (2–6). While the prospective phase III trial RTOG 0631 demonstrated that SBRT can be safely and accurately delivered to 2 contiguous vertebral levels using image-guided radiotherapy (IGRT) (7), there are limited data evaluating the feasibility of SBRT to ≥3 spinal levels. One concern is that small setup errors (particularly rotational) could be magnified when treating ≥3 levels resulting in tumor undercoverage or increased dose to critical neural structures such as the spinal cord and cauda equina (8). However, modern treatment techniques such as 6-degree-of-freedom (6-DOF) couch tops, full-body immobilization, and cone-beam computed tomography (CBCT) may allow reproducible and accurate SBRT delivery within 1 mm even when targeting ≥3 contiguous vertebral levels (9). If so, the LC, symptom palliation, and decreased toxicity benefits of SBRT could be extended to appropriately selected patients with ≥3 contiguous involved levels who currently are frequently offered conventional EBRT. To demonstrate the feasibility of multilevel spine SBRT, we compared LC, dosimetric, and toxicity data of patients who received treatment to ≥3 contiguous spinal levels to those receiving SBRT to just 1–2 levels.

## Methods

### Study Design

We retrospectively evaluated patients with spine metastases treated with SBRT at a single tertiary care institution from 2013 to 2019. Patients who underwent surgical resection or stabilization before SBRT were eligible for inclusion. Patients with cervical, thoracic, lumbar, or sacral metastases were included. Pertinent patient demographic and disease characteristics were collected including, but not limited to, age, gender, performance status defined using Karnofsky Performance Status (KPS), tumor histology, systemic disease control, receipt of concurrent systemic therapy, site of spine metastases, Spinal Instability Neoplastic Score (SINS), and Bilsky grade. Concurrent systemic therapy was defined as systemic therapy given within 2 weeks of RT. For patients undergoing surgery, the type of surgical intervention was documented. Pertinent information regarding RT was collected including, but not limited to, prescribed dose and fractionation and the size of the planning target volume (PTV). Biologically effective doses (BEDs) were calculated using α/β ratios of 10 for the tumor and 2 for the spinal cord and cauda equina. Using these BED values, dosimetric data were collected including, but not limited to, the minimum and maximum doses to the PTV, dose received by 95% of the PTV (D95%) and D90%, and max point doses to the spinal cord and cauda equina, where applicable. This study was approved by our institutional review board.

### Treatment

All patients underwent spine magnetic resonance imaging (MRI) prior to surgery or spine SBRT (General Healthcare 1.5 Tesla MR, Chicago, IL, USA, or Siemens Healthcare 1.5T or 3T MR, Erlangen, Germany). Patients who underwent surgery completed additional postoperative MRI and received a computed tomography (CT) myelogram on the same day as simulation for radiation planning. Patients were simulated in the supine position without intravenous contrast (GE Discovery CT590RT, Chicago, IL, USA). A thermoplastic frameless mask was used for immobilization of the cervical spine. A stereotactic body frame and vac loc bag were used to immobilize patients for the treatment of the thoracic, lumbar, and/or sacral spine. For accurate delineation of target volumes and organs at risk, spine MRI sequences, including but not limited to, axial and sagittal T1 postcontrast and T2, were fused to the CT simulation. For patients receiving postoperative SBRT, both preoperative imaging and postoperative imaging were fused as was the CT myelogram.

The clinical target volume (CTV) included gross osseous and extraosseous disease plus bony anatomy at risk of harboring microscopic disease as per consensus contouring guidelines for both postoperative and definitive spine SBRT (10, 11). Regardless of the number of treated levels, the PTV was equivalent to the CTV (no expansion). The spinal cord was delineated on the postsurgery CT myelogram or the MRI T2 axial series for nonoperative patients with a 2-mm circumferential expansion to create a planning risk volume (PRV) avoidance structure. SBRT was prescribed at a dose of 14–35 Gy in 1–5 fractions and normalized with the goal that at least 95% of the PTV received the prescription dose. Spinal cord constraints were based on the The American Association of Physicists in Medicine (AAPM) TG 101 recommendations appropriate for the chosen treatment fractionation (12). Volumetric modulated arc therapy with coplanar arcs utilizing 6 or 10 megavoltage beams was predominantly used for treatment planning (Eclipse version 16, Varian Medical Systems, Palo Alto, CA, USA). Treatments were delivered on a linear accelerator (Varian TrueBeam, Palo Alto, CA, USA) with 6-DOF and daily CBCT to minimize setup uncertainty.

### Study Endpoints

The primary study endpoint was the rate of LC at 1 year. LC was assessed using follow-up MRIs of the treated spine that occurred every 3 months post-SBRT. Patients were also followed with clinic appointments at that interval with a detailed history and physical exam. Radiographic tumor response was assessed as per SPIne response assessment in Neuro-Oncology recommendations (13). Serial MRI concerning radiographic evidence of progression was retrospectively reviewed for confirmation of local failure (LF) by two independent neuroradiologists. Serial imaging was routinely used to differentiate between progression and pseudoprogression based upon evolution over time. Where available, perfusion sequences were also utilized. Secondary endpoints included progression-free survival (PFS), overall survival (OS), and rates of acute and chronic toxicity. PFS was defined as the time from the start of SBRT to the time of progression, death, or last follow-up. OS was defined as the time from the start of SBRT to the time of death or last follow-up. Acute and chronic toxicities were defined as per the National Cancer Institute Common Terminology Criteria for Adverse Events v5.0. The proportion of plans where 95% of the PTV was covered by at least 95% of the prescription volume was also assessed to investigate the feasibility of multilevel spine SBRT.

### Statistical Analysis

Patients were stratified into two groups. Group A consisted of patients who received SBRT to 1–2 contiguous levels, while group B included patients with ≥3 contiguous treated levels. Continuous and discrete variables were compared between groups using the Mann–Whitney U and Pearson’s chi-square tests, respectively (14). Median follow-up was calculated using the reverse Kaplan–Meier (KM) method. KM curves were used to assess the endpoints of OS, PFS, and LC. Hazard ratios (HRs) and 95% confidence intervals (CIs) were calculated with a two-sided significance threshold of 0.05. For analysis, dosimetric variables were dichotomized by the median. Univariate analysis (UVA) was performed using a proportional hazards model to assess for a significant relationship between clinically relevant variables and improved LC. Cox proportional hazards modeling multivariate analyses (MVAs) were then performed using variables that achieved significance on UVA and/or clinically relevant factors to examine for an association with increased likelihood of LF. All statistical analyses were performed using SPSS Statistics version 27 (IBM, Armonk, NY, USA).

## Results

### Patient Demographic and Treatment Characteristics

Overall, 165 patients with 194 metastatic spine sites treated with SBRT with a median follow-up of 15 months (range: 1–72 months) were identified (**Table 1**). Among them, 54% of the patients were men, the median patient age at treatment was 61 (range: 24–93) years, and 93% of patients had a KPS >70. In this study, 32% of patients underwent separation surgery. In addition, 24% and 8% of patients underwent vertebrectomy and laminectomy, respectively. SBRT was offered for improved LC (through delivery of higher BED) in radioresistant histology, pain, ablation of oligometastatic disease, or oligoprogression in 44%, 36%, 11%, and 9% of patients, respectively. The thoracic spine (65%) was the most treated site. A total of 113 patients (68%) received treatment at 1–2 contiguous levels, and 52 (32%) received SBRT at 3–7 contiguous levels. The most prescribed regimen was 27 Gy in 3 fractions. The most common histologies treated were renal cell carcinoma (RCC) (31%), non-small cell lung cancer (NSCLC) (13%), and breast cancer (11%). Moreover, 45% of patients received concurrent systemic therapy, which was most frequently chemotherapy (20%) or immunotherapy (13%).

**Table 1:** Patient demographic and treatment characteristics stratified by the number of contiguous treated spinal levels.

Variable	All	1–2 levels	3–7 levels	p value
Number of Courses	194	140	54	
Number of Patients	165	113	52	
Median Follow-up (Months)	15 (1–72)	15 (2–72)	15 (1–63)	0.873
Median Age (Years)	61 (24–93)	61 (24–87)	61 (24–93)	0.986
Gender				0.534
Men	104 (54%)	72 (51%)	32 (59%)	
Women	90 (46%)	68 (49%)	22 (41%)	
Karnofsky Performance Status				0.294
≥70%	180 (93%)	128 (91%)	52 (96%)	
<70%	14 (7%)	12 (9%)	2 (4%)	
Surgery				**0.001**
Vertebrectomy	47 (24%)	24 (17%)	23 (42%)	
Laminectomy	16 (8%)	6 (4%)	10 (19%)	
None	131 (68%)	110 (79%)	21 (39%)	
Median Prescription Dose (Gy)	27 (14–35)	27 (15–35)	27 (14–28)	0.700
Median Prescription BED* (Gy)	51 (33–60)	51 (37.5–60)	51 (33–56)	0.279
Median PTV Minimum BED* (Gy)	30 (11–52)	31 (12–52)	28 (11–44)	0.262
Median PTV Mean BED* (Gy)	54 (34–69)	54 (37–69)	54 (34–63)	0.393
Median PTV D95% BED* (Gy)	51 (26–62)	51 (33–62)	51 (26–52)	0.572
Median PTV D90% BED* (Gy)	52 (30–64)	52 (36–64)	52 (30–54)	0.986
Median Spinal cord Max BED** (Gy)	50 (10–114)	46 (9–99)	57 (10–114)	0.326
Median Cauda Equina Max BED** (Gy)	72 (5–144)	79 (5–144)	58 (31–94)	0.327
PTV Coverage				0.078
≥95% PTV Coverage by ≥95% Prescription Dose	182 (94%)	134 (96%)	48 (89%)	
Not Met	12 (6%)	6 (4%)	6 (11%)	
Fractionation				**0.007**
Single	31 (16%)	28 (20%)	3 (6%)	
Multi	163 (84%)	112 (80%)	51 (94%)	
Median Treated Volume (cc)	65 (6–670)	52 (6–486)	150 (29–670)	**0.001**
Histopathology				**0.040**
Breast	22 (11%)	13 (9%)	9 (17%)	
Renal Cell Carcinoma	60 (31%)	45 (32%)	15 (28%)	
Sarcoma	19 (9%)	12 (9%)	7 (13%)	
Thyroid	13 (7%)	11 (8%)	2 (2%)	
Gastrointestinal	19 (9%)	16 (11%)	3 (6%)	
Non-small Cell Lung Cancer	26 (13%)	21 (15%)	5 (9%)	
Neuroendocrine	4 (2%)	4 (3%)	0 (0%)	
Prostate	9 (5%)	3 (2%)	6 (12%)	
Melanoma	7 (3%)	6 (4%)	1 (1%)	
Head and Neck Squamous Cell Carcinoma	9 (5%)	3 (2%)	6 (12%)	
Metastatic Pituitary	1 (1%)	1 (1%)	0 (0%)	
Metastatic Paraganglioma	3 (2%)	3 (2%)	0 (0%)	
Bladder	1 (1%)	1 (1%)	0 (0%)	
Adenoid Cystic Carcinoma	1 (1%)	1 (1%)	0 (0%)	
Vertebral Site				0.572
Cervical	22 (10%)	17 (12%)	5 (9%)	
Cervical-Thoracic	8 (5%)	4 (3%)	4 (7%)	
Thoracic	103 (53%)	68 (48%)	35 (65%)	
Lumbar	45 (23%)	40 (29%)	5 (9%)	
Thoracic-Lumbar	7 (3%)	3 (3%)	4 (7%)	
Sacral	9 (6%)	8 (5%)	1 (3%)	
Concurrent Systemic Therapy				0.540
Chemotherapy	38 (20%)	23 (16%)	15 (28%)	
Immunotherapy	26 (13%)	20 (14%)	6 (15%)	
Targeted Therapy	20 (10%)	16 (11%)	4 (7%)	
Radioactive Iodine	3 (2%)	3 (2%)	0 (0%)	
None	107 (55%)	78 (56%)	29 (54%)	
Systemic Disease Control				**0.001**
Stable	153 (79%)	115 (82%)	38 (70%)	
Progression	41 (21%)	25 (18%)	16 (30%)	
Spinal Instability Neoplastic Score				**0.001**
</=7	134 (69%)	107 (76%)	27 (50%)	
>7	60 (31%)	33 (24%)	27 (50%)	
Bilsky Grade				**0.001**
1a/b	118 (61%)	98 (70%)	20 (37%)	
1c	24 (13%)	20 (14%)	4 (7%)	
2	29 (14%)	15 (10%)	14 (26%)	
3	23 (12%)	7 (6%)	16 (30%)	
Reason for SBRT				
Oligometastasis	21 (11%)	10 (7%)	11 (20%)	
Oligoprogression	17 (9%)	17 (12%)	0 (0%)	
Radioresistant Histology	86 (44%)	70 (50%)	16 (30%)	
Pain	70 (36%)	43 (31%)	27 (50%)	

BED, biologically effective dose; *BED10; **BED2; PTV, planning target volume. bold values achieved statistical significance.

With respect to patients with 1–2 treated levels, patients with ≥3 irradiated levels were more likely to have received a surgical intervention (61% vs. 21%, p = 0.001), have received fractionated SBRT rather than single-fraction radiosurgery (94% vs. 80%, p = 0.007), have a larger PTV (150 vs. 52 cc, p = 0.001), have progressive systemic disease (30% vs. 18%, p = 0.001), and have an SINS score greater than 7 (50% vs. 25%, p = 0.001) and a higher Bilsky grade (p = 0.001). Patients with ≥3 irradiated levels were more likely to have breast, prostate, head and neck, or sarcoma histologies and less likely to have NSCLC or RCC (p = 0.04). There was no significant difference between groups by gender, age, receipt of systemic therapy, or performance status.

### Local Control and Survival Outcomes

**Figure 1:**
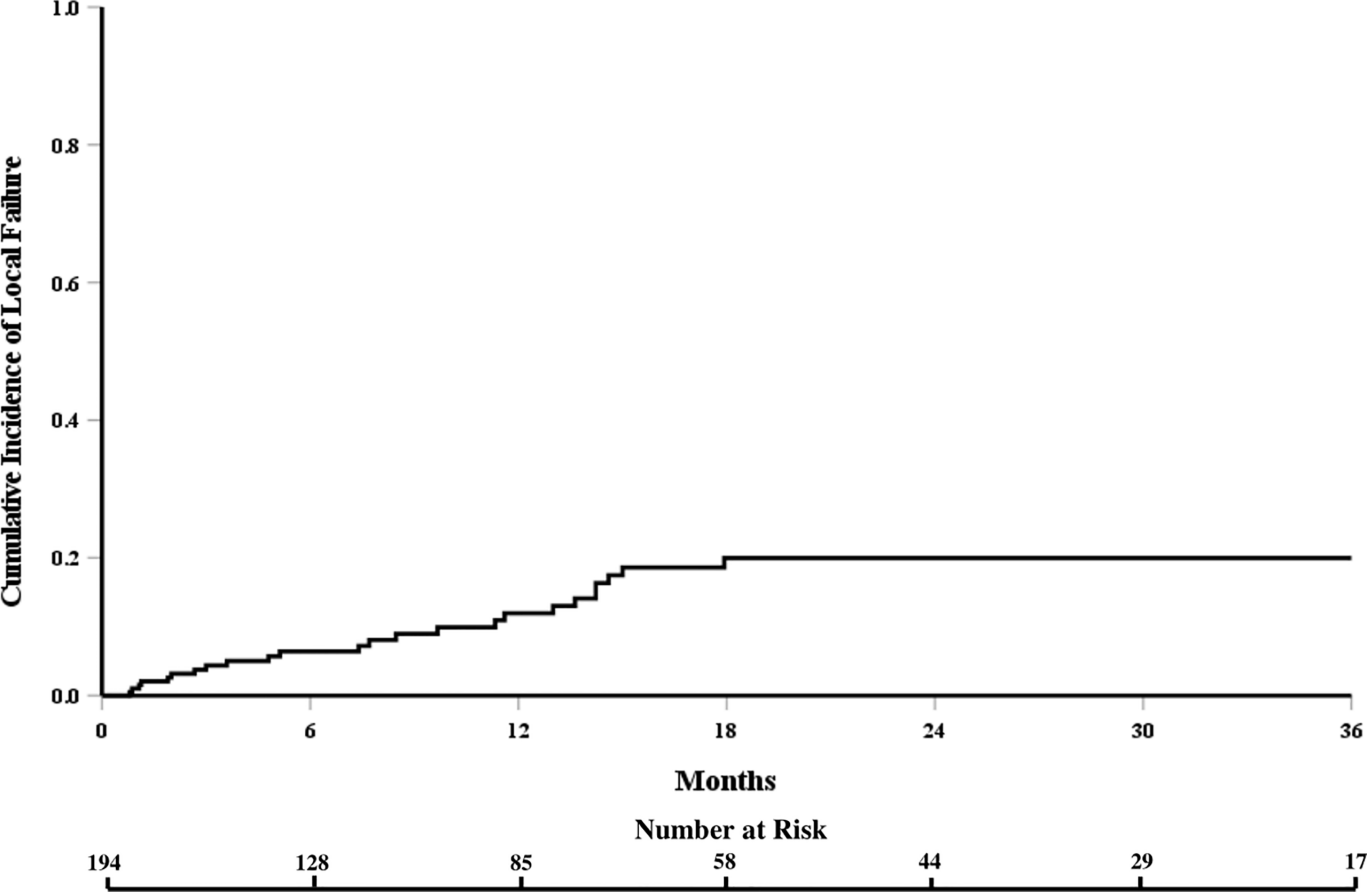
Cumulative incidence of local failure for the entire cohort of patients receiving spine stereotactic body radiation therapy.

**Figure 2:**
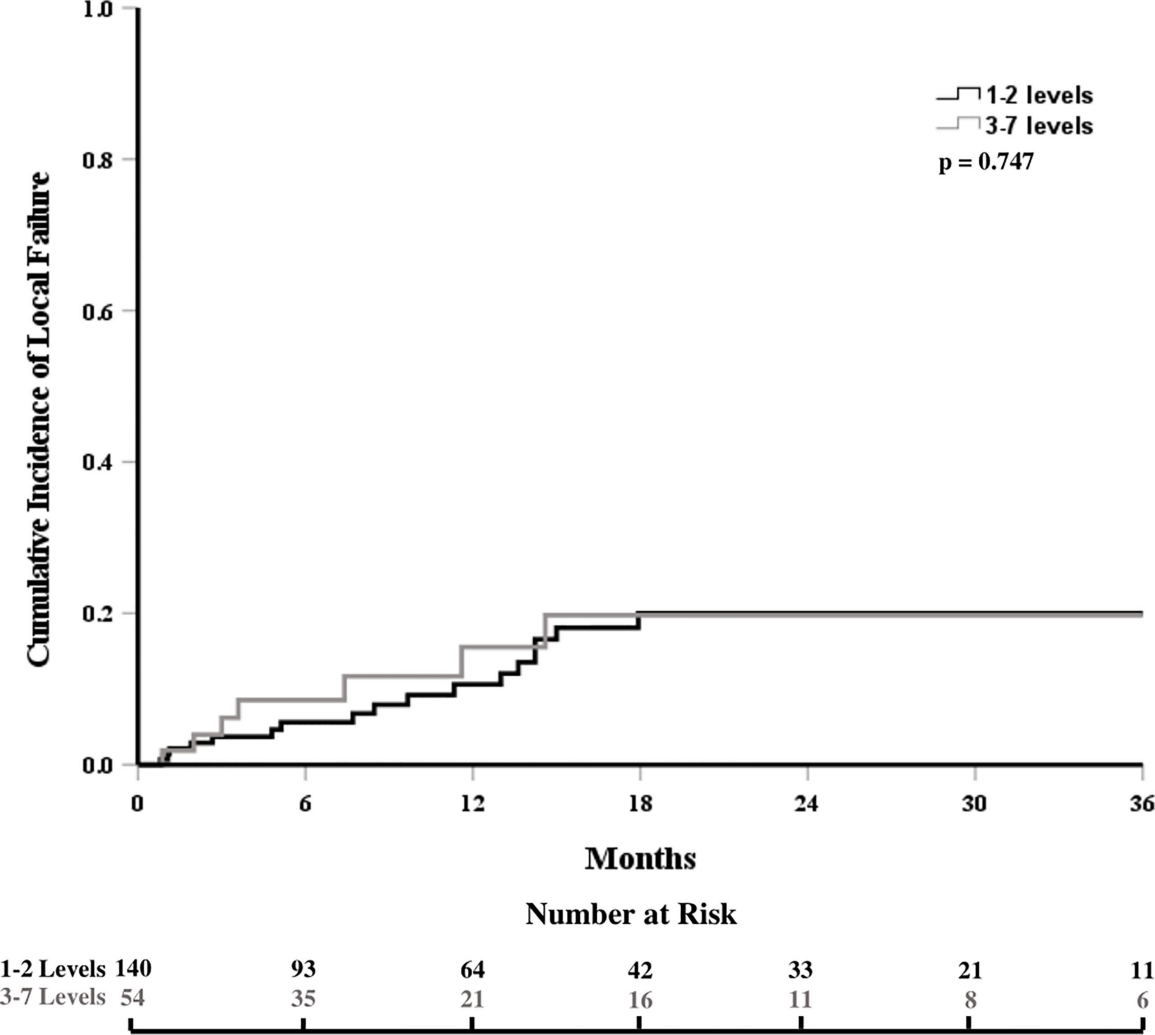
Cumulative incidence of local failure stratified by the number of contiguous treated levels.

The cumulative incidence of LF for the whole cohort at 6, 12, and 18 months was 6%, 12%, and 18%, respectively (**Figure 1**). When stratified by the number of contiguous levels treated, there was no difference in 1-year rates of LF (11% for 1–2 levels vs. 16% for ≥3 levels, p = 0.747, **Figure 2**). In patients who experienced LF, time to failure did not differ between groups (9.8 for 1–2 levels vs. 7.6 months for ≥3, p = 0.873). On UVA, no demographic or disease characteristics significantly predicted improved LC in patients with ≥3 treated levels (**Table 2**). On MVA, male gender [HR 2.734 (CI: 1.047–7.142), p = 0.040] and stable systemic disease at the time of SBRT were associated with better LC [HR 4.154 (CI: 1.667–10.355), p = 0.002] for these patients with multiple contiguous treated levels (**Table 3**). No other demographic or disease variables achieved significance. The 1-year OS and PFS for this cohort were 62% and 56%, respectively.

**Table 2:** Univariate analysis examining for variables associated with improved local control in patients with ≥3 contiguous treated levels using a proportional hazards model.

Variable	1-Year Local Control	p value
Age		
≤60	89%	0.882
>60	88%	
Gender		
Men	91%	0.278
Women	75%	
Surgery		
Vertebrectomy	80%	0.148
Laminectomy	90%	
None	95%	
Prescription BED*		
≥51.3Gy	84%	0.869
<51.3Gy	87%	
PTV Minimum BED*		
≥28Gy	82%	0.834
<28Gy	87%	
PTV Mean BED*		
≥55.13Gy	81%	0.492
<54.13Gy	90%	
PTV D90% BED*		
≥52Gy	82%	0.654
<52Gy	89%	
PTV D95% BED*		
≥51.3Gy	82%	0.632
<51.3Gy	90%	
PTV Coverage by Prescription Dose		
≥95%	85%	0.706
<95%	83%	
Treated Volume		
<150cc	90%	0.292
≥150cc	79%	
Systemic Disease Control		
Stable	92%	0.090
Progressive	54%	
Spinal Instability Neoplastic Score		
≤7	96%	0.122
>7	69%	
Bilsky		
Grade 1a–c	96%	0.171
Grade 2–3	71%	
Fractionation		
Single	67%	0.270
Multi	86%	
Histopathology		
Renal Cell Carcinoma	93%	0.329
Rest	81%	

BED*, biologically effective dose 10; PTV, planning target volume.

**Table 3:** Multivariate analysis examining for variables associated with improved local control in patients with ≥3 contiguous treated levels using a proportional hazards model.

Variable	Hazard Ratio	95% Confidence Interval	p value
Age	1.325	0.562–3.125	0.520
Gender	2.734	1.047–7.142	**0.040**
Surgery vs. None	1.708	0.486–6.007	0.404
Prescribed BED*	0.357	0.084–1.519	0.163
Planning Target Volume D90% BED*	1.120	0.304–4.132	0.865
Treatment Volume	0.693	0.269–1.787	0.448
Histopathology	1.022	0.946–1.105	0.578
Spinal Instability Neoplastic Score	1.442	0.962–2.162	0.077
Bilsky	1.234	0.630–2.416	0.539
Systemic Disease Control	4.154	1.667–10.355	**0.002**

BED*, biologically effective dose 10; CI, confidence interval; HR, hazard ratio; D90%, minimum dose received by 90% of the planning target volume; D95%, minimum dose received by 95% of the planning target volume. bold values achieved statistical significance.

### Feasibility of Treatment Delivery and Dosimetry

For patients with ≥3 vs. 1–2 contiguous treated levels, the median prescribed dose (27 vs. 27 Gy), prescribed dose to the tumor in BED (51 vs. 51 Gy), minimum dose to the PTV in BED (28 vs. 31 Gy), mean dose to the PTV in BED (54 vs. 54 Gy), D95% in BED (51 vs. 51 Gy), and D90% in BED (52 vs. 52 Gy) were similar, with all p values nonsignificant. The median max spinal cord point dose in BED was similar between patients with ≥3 vs. 1–2 contiguous treated levels (57 vs. 46, p = 0.326). The median max cauda point dose in BED was similar between patients with ≥3 vs. 1–2 contiguous treated levels (58 vs. 79, p = 0.327). For patients with ≥3 contiguous treated levels, the range of max point spinal cord doses, by BED, was 10–114 Gy, and the range of max point cauda equina doses was 31–94 Gy. The maximum spinal cord point BED of 114 Gy occurred in the treatment of a patient with a high Bilsky grade who was not able to undergo spinal separation surgery. Coverage of at least 95% of the PTV with at least 95% prescription dose was similar between groups (96% 1–2 levels vs. 89% ≥3 levels, p = 0.078). Of note, 10 patients had 4–7 contiguous levels treated with a median follow-up of 15.6 months (range: 1–52) (**Table 4**). In these most complex treatment scenarios, 70% of treatment plans resulted in coverage of at least 95% of the PTV with at least 95% of the prescription dose while still respecting the constraints of organs at risk. In patients with ≥3 treated levels, early recurrences were found in several patients with high-grade sarcomas, chondrosarcoma, and triple-negative breast cancer.

**Table 4:** Plan and treatment delivery characteristics for patients with 4–7 contiguous treated levels.

Case	Site	Dose (Gy/fx)	NTL	Minimum dose (BED10)	Mean dose (BED10)	D90% (BED10)	SC/CE max dose (BED2)	Coverage	Number of CBCT/course
1	T	25/5	4	35.4	43.1	38.4	114.6	≥95%	5
2	C	27.5/5	4	19.8	44.5	42.0	28.13	<95%	6
3	T	25/5	4	29.6	39.3	38.2	66.72	≥95%	10
4	T-L	27/3	5	14.9	51.3	49.2	32.06	<95%	5
5	L	27/3	5	36.7	54.1	51.9	56.78	≥95%	6
6	T	25/5	5	13.1	40.6	36.5	21.53	<95%	6
7	C-T	25/5	5	10.8	41.0	38.8	50.03	≥95%	6
8	C-T	25/5	6	16.5	40.8	38.3	10.27	≥95%	9
9	T	25/5	6	14.8	40.0	37.5	30.48	≥95%	7
10	T	25/5	7	28.9	39.4	38.2	71.7	≥95%	7

BED, biologically effective dose; C, cervical spine; CBCT, cone beam CT; CE, cauda equina; T, thoracic spine; L, lumbar spine; NTL, number treated levels; SC, spinal cord.

### Acute and Chronic Toxicities

There was no significant difference between the two groups in terms of acute toxicities (18% vs. 21%), which were limited to grade 1–2 fatigue, grade 1 dermatitis, grade 1 nausea, and grade 1 pain flare. There were no grade 3 or higher acute events. Pain flare incidence was 5.5% in the whole cohort in which patients required steroids ± opioids. Regarding chronic toxicity, of the 131 courses of definitive spine SBRT without surgical intervention, 11 cases (8.4%) presented with progressive/new vertebral compression fracture (VCF) over a median follow-up of 15 months. There was no difference between patients with 1–2 (8%) and ≥3 treated levels (10%) in terms of progressive/new VCF (OR 1.18, CI 0.236–5.902, p = 0.839). There were no events of radiation-induced myelopathy, radiculopathy, or plexopathy in either group, and there were no reported grade 3 and above late toxicities.

## Discussion

This study represents one of the first analyses to suggest that SBRT to ≥3 contiguous spinal levels may be feasible and similarly safe and effective to treatment to 1–2 levels. These findings have strong clinical relevance because a recent phase III trial demonstrated improved treatment response with SBRT with respect to conventional EBRT (15), but patients with multilevel disease have been previously considered to be suboptimal candidates for SBRT due to feasibility concerns. Regarding feasibility, coverage of at least 95% of the PTV with at least 95% prescription dose was achievable in 89% of patients with ≥3 treated contiguous levels, no different from the rate seen for patients with 1–2 treated levels. While repositioning with reimaging was required for some fractions, particularly for patients with 4 or more involved levels, there were no issues with treatment delivery.

Additionally, treatment for these complex patients was similarly effective to SBRT to 1–2 levels in both our patients and historical controls. Multiple studies have demonstrated that 1-year control rates with spine SBRT of 71%–97% may be superior to tumor control with conventional radiotherapy (2–5, 16–19). Our 1-year LF rate of 12% for the entire cohort (1–7 treated levels) was similar to these historical controls. Most importantly, our 1-year LF rate for those patients with ≥3 treated levels was just 16%, which was not statistically different from the rate of LF for patients receiving treatment to 1–2 levels. While patients with ≥3 treated levels were more likely to undergo surgery or have breast or prostate histologies, this imbalance likely does not explain the excellent rates of LC in this subcohort, as these variables did not significantly predict LC on UVA or MVA. While infrequent, the failures in this subgroup were primarily driven by the preponderance of patients with extremely radioresistant histologies such as sarcoma. These findings are similar to those from Beeler et al. (20) showing 1-year LC rates for single-level and multilevel SBRT of 95% vs. 85% (p = 0.11). On MVA, progressive systemic disease at the time of SBRT was significantly associated with inferior LC. This relationship may be because poor systemic disease control portends a more treatment-resistant disease process. Additionally, female gender was significantly associated with inferior LC. Given the almost borderline p value for this variable, these results are likely attributable to chance.

Notably, SBRT to ≥3 levels did not result in increased acute or chronic toxicity with respect to our patients with fewer treated levels or to historical controls. While SBRT is known to increase the risk of VCF to about 15% and radiation-induced myelopathy can also be a concern (16, 21–23), the risk of progressive/new VCF in our patients with ≥3 levels was just 9.5% with a 0% rate of radiation-induced myelopathy. In our study, there was no statistically significant difference between groups in terms of acute or late toxicities and there were no high-grade toxicities regardless of the number of contiguous treated levels. With this excellent durable LC and low incidence of side effects, multilevel contiguous SBRT may be a good option for appropriately selected patients with spine metastases.

Some limitations of this approach and this analysis should be considered. Due to differences in group size, match-pair analysis was not feasible, and there may be unaccounted for differences between groups. However, besides systemic disease control and gender, no variables were significant predictors of LF on MVA, suggesting that the inability to conduct match-pair analysis may have a relatively minimal impact. Spine SBRT, particularly multilevel, poses unique barriers to reliable daily setup and accurate treatment delivery. A sharp dose falloff is required close to critical structures with the potential for around 2-Gy perturbation to the Dmax delivered to the spinal cord if there is even a 1-mm vertical misalignment (9). The utilization of a headrest for patient comfort with a thermoplastic head and neck mask for immobilization for cervical and upper thoracic spine SBRT can result in an air gap with cervical and upper thoracic spine curvature being difficult to reproduce on a daily basis (9). However, modern equipment such as 6-DOF couches can correct these translational and rotational errors (24, 25). This capability, in combination with full-body immobilization and CBCT, has led to the achievement of submillimeter accuracy for treatment delivery in about 98% of SBRT cases, particularly for treatments delivered to T2–S5 (9). Thus, at our institution, we used a 0-mm expansion from CTV to PTV and 2-mm PRV expansion around the spinal cord/cauda equina for both postoperative and definitive spine SBRT patients. However, another retrospective study noted mean translational errors of 0.5 mm with a standard deviation of 0.5 mm for multilevel spine SBRT (26). Thus, although our rates of LF were excellent despite no PTV expansion, we acknowledge that PTV expansions should be considered depending upon institutional comfort with setup reproducibility. Additionally, the best candidates for SBRT with multilevel disease are likely to be well-performing patients eligible for debulking and with excellent systemic therapy options; otherwise, the potential benefit in improved durable LC with this approach, with respect to standard palliative techniques, may not be meaningful. Additional limitations primarily stem from the study’s retrospective nature and include potential patient selection biases.

In summary, in appropriately selected patients, spine SBRT to 3–7 contiguous vertebral levels is feasible and may offer high rates of LC with minimal toxicity. As a result, the benefits of spine SBRT may be extendable to patients with multilevel disease who have been considered more appropriate for conventional EBRT. A prospective evaluation of this approach is warranted.

## Data Availability Statement

The raw data supporting the conclusions of this article will be made available by the authors without undue reservation.

## Ethics Statement

The studies involving human participants were reviewed and approved by The Ohio State University Institutional Review Board. Written informed consent for participation was not required for this study in accordance with the national legislation and the institutional requirements.

## Author Contributions

KD, DMB: first authors. RP, AO, EB, DB, AA, EC, WM, PM, ET, HL, JG, RR, EM, TS, RL, AC, and JE: contributors. JP: senior author. All authors contributed to the article and approved the submitted version.

## Conflict of Interest

The authors declare that the research was conducted in the absence of any commercial or financial relationships that could be construed as a potential conflict of interest.

## Publisher’s Note

All claims expressed in this article are solely those of the authors and do not necessarily represent those of their affiliated organizations, or those of the publisher, the editors and the reviewers. Any product that may be evaluated in this article, or claim that may be made by its manufacturer, is not guaranteed or endorsed by the publisher.
